# Mendelian randomization analysis reveals causal effects of metformin on bipolar disorder risk

**DOI:** 10.1097/MD.0000000000045112

**Published:** 2025-10-24

**Authors:** Zhitao Li, Lili Wang, Xinyu Sun, Yichuan Lin

**Affiliations:** a Psychological Clinic, Quanzhou First Hospital Affiliated to Fujian Medical University, Quanzhou, Fujian Province, China; b Department of Psychiatry, The Third Hospital of OuanZhou, Quanzhou, Fujian Province, China.

**Keywords:** bipolar disorder, mendelian randomization analysis, metformin

## Abstract

Metformin, as a drug for treating type 2 diabetes, has been found to have a positive impact on mental health. This study will assess the potential causal relationship between metformin exposure and the risk of bipolar disorder (BD). We used publicly available genome-wide association studies statistical data to conduct 2-sample bidirectional Mendelian randomization (MR) analysis to determine the causal relationship between genetic susceptibility to metformin and BD risk. Genome-wide significant single nucleotide polymorphisms, that are associated with metformin use were selected as the instrumental variables. To determine the causal relationship between metformin exposure and BD, MR analysis was conducted, employing methods such as instrumental variable weighting. We applied complementary methods, including weighted median, weighted mode, simple mode, MR-Egger regression, and MR-pleiotropy residual sum and outlier to detect and correct for the effect of horizontal pleiotropy. Cochran *Q* statistics were used to assess instrument heterogeneity. The leave-one-out method was conducted for sensitivity analysis. The instrumental variable weighting analysis demonstrated that there was a significant causal relationship between the metformin exposure and BD risk [odds ratio [OR] (95% confidence interval [CI]) = 0.032 (0.002–0.593), *P* = .021] (ebi-a-GCST003724), [OR (95% CI) = 1.452E‐04 (1.442E‐07 to 1.463E‐01), *P* = .012] (ieu-a-800), [OR (95% CI) = 0.33 (0.27–0.404), *P* = .000] (ieu-a-808). The sensitivity analysis revealed no heterogeneity in the individual results (*P* > .05), and no significant publication bias. This study reveals that metformin may serve as a protective factor for BD, providing new theoretical basis for the application of metabolic intervention in the prevention and treatment of mental illness.

## 1. Introduction

Bipolar disorder (BD) is a severe mental illness characterized by extreme emotional fluctuations, with clinical manifestations of alternating manic (or hypomanic) and depressive episodes. The global prevalence rate is about 2.4%, and it is one of the main causes of disability worldwide.^[1,2]^ Its complex etiology involves multiple system interactions such as genetics, metabolism, and neuroinflammation.^[3]^ Although existing treatment methods including mood stabilizers and antipsychotic drugs can partially control symptoms, about 40% of patients have poor response to drugs. Long-term medication often accompanies significant metabolic side effects (such as weight gain and insulin resistance), which may further worsen the disease prognosis.^[4–6]^ It is worth noting that epidemiological studies have confirmed that the morbidity of metabolic syndrome in patients with BD is as high as 35% to 50%, much higher than the general population (about 20%–25%).^[7,8]^ This high prevalence not only exacerbates cardiovascular risk, but also may directly affect the disease process through the “metabolic-brain axis” mechanism (such as neuroinflammation, mitochondrial dysfunction),^[7,9]^ forming a vicious cycle of “metabolic abnormalities–worsening symptoms–treatment resistance.”

Metformin, as a drug for treating type 2 diabetes, has gradually become a research hotspot in diagnosis and treatment of mental diseases in recent years due to its potential neuroprotective effects and its ability to regulate metabolic disorders.^[10,11]^ Basic research have shown that metformin not only improved insulin sensitivity, but may also exerted neuroprotective effects by reducing microglial activation, promoting brain-derived neurotrophic factor (BDNF) release, and enhancing synaptic plasticity.^[12–14]^ Clinical observational studies have also found that patients with BD who use metformin exhibit lower disease recurrence rates and more stable emotional states.^[15]^ However, there is no literature report on whether metformin has therapeutic effects on BD. Traditional research methods, such as cohort study or randomized controlled trial are limited by cost and ethical issues or other factors, and can not give a clear answer, leading to a significant gap in the existing evidence.

Mendelian randomization (MR) method simulates the process of random grouping by using genetic variation as an instrumental variable (IV), providing new ideas for analyzing the causal relationship between exposure and outcome.^[16]^ In recent years, genome-wide association studies (GWAS) have identified multiple genetic loci associated with the pharmacology of metformin and the risk of BD, laying the foundation for MR analysis. MR analysis has become an established tool for exploring causal relationships in characterizing disease etiology. MR analysis leverages genetically determined variation in exposure (through single nucleotide polymorphism [SNPs] as IVs) to estimate causal effects on disease risk, greatly reducing susceptibility to confounding factors.^[17]^ This method utilizes the random classification of genetic variations during meiosis, effectively mimicking natural randomized controlled trials and minimizing bias in reverse causality and unmeasured confounding factors. In our study, we will use MR analysis to evaluate the potential causal relationship between exposure to metformin and the risk of BD.

## 2. Materials and methods

### 2.1. Study design

**Figure 1.**
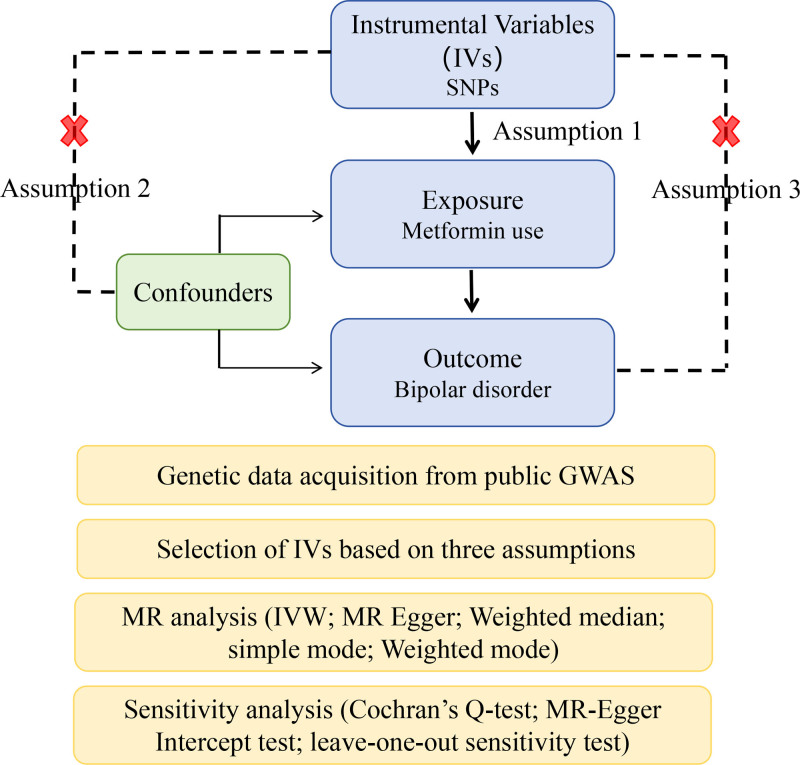
Schematic of a 2-sample MR study. GWAS = genome-wide association studies, IVs = instrumental variables, MR = Mendelian randomization, SNPs = single nucleotide polymorphisms.

MR analysis of 2 samples was adopted to evaluate the relationship between metformin and BD. The selected IVs should meet 3 important assumptions (Fig. 1): IVs are strongly correlated with metformin; IVs are not associated with any confounding factors; and IVs are not directly correlated with bipolar disorder through factors other than metformin.

### 2.2. Data source

In our study, the exposure data was sourced from the UK Biobank (https://gwas.mrcieu.ac.uk/datasets/). The metformin exposure GWAS (ID: ukb-b-14609) is derived from electronically recorded primary care prescriptions in UK Biobank (nonself reported). Cases were defined as individuals with ≥1 metformin prescription (ATC: A10BA02), excluding insulin users. This data source minimizes recall bias and has been validated against NHS records. Extract the result data of metformin exposure and multiple BD studies from the EBI database: metformin (N = 462,933), BD (ebi-a-GCST003724; N = 34,950), BD (ieu-a-800; N = 16,731), BD (ieu-a-808; N = 3049), as shown in Table 1. Due to the data being sourced from the IEU Open GWAS project, it can be downloaded for free (https://gwas.mrcieu.ac.uk/), no ethical approval or participant consent is required.

**Table 1:** Detailed information about the aggregated GWAS results.

GWAS ID	Trait	Author	Consortium	Sample size	Number of SNPs
ukb-b-14609	Metformin	Ben Elsworth	MRC-IEU	462,933	9,851,867
ebi-a-GCST003724	Bipolar disorder	Hou L	NA	34,950	9,483,147
ieu-a-800	Bipolar disorder	Sklar P	PGC	16,731	108,835
ieu-a-808	Bipolar disorder	Smith EN	NA	3049	699,461

The 3 BD GWAS were selected to ensure: Maximized power via PGC-BD (largest available BD GWAS); Population diversity via FinnGen (Finnish) and iPSYCH (Danish); and Phenotype rigor (all clinician-diagnosed per DSM/ICD).

UK Biobank BD data was excluded due to smaller sample size and potential misclassification. PGC-BD served as the primary dataset.We have confirmed that there was no overlap between different samples. Exposure: UK Biobank-only cohort (ukb-b-14609); Outcomes: ebi-a-GCST003724 (PGC-BD): Independent international consortium; ieu-a-800 (FinnGen): Finnish national cohort; ieu-a-808 (iPSYCH): Danish psychiatric registry cohort. This satisfies the 2-sample MR independence assumption.

### 2.3. Selection of the genetic instruments

The following inclusion criteria guided our selection of the IVs: First, SNPs should have a genome-wide significance level (*P* < 5 × 10^‐8^), which strongly indicates a genetic association with exposure; Second, then set parameters (*r*^2^ < 0.001 and window size = 10,000) to exclude SNPs with strong linkage disequilibrium and differentiate their independence; Third, set a threshold value of *F* > 10 as a reliable and effective IV to prevent a weak instrument bias.^[18,19]^ Furthermore, we found no confounding SNPs by searching for SNP information in web: https://ldlink.nih.gov/?tab=ldtrait, GWAS catalog, and PubMed.

### 2.4. Statistical analysis

The inverse variance weighted (IVW) method was chosen as the main approach to evaluate the causal relationship between metformin and BD. Four additional evaluation techniques, including MR-Egger regression, weighted median, simple mode, and weighted mode were used as supplements to IVW.^[20,21]^ Cochran *Q* statistics were used to reflect instrument heterogeneity, *P* < .05 was considered heterogeneity.^[22]^ Horizontal pleiotropy was evaluated using MR-Egger intercept test and MR-pleiotropy residual sum and outlier (MR-PRESSO) global test, *P* < .05 was considered pleiotropy.^[23]^ We also used the MR-PRESSO method to identify potential outliers. If outliers were found, we would exclude them and perform MR analysis again. Finally, we conducted a sensitivity analysis on the results using the leave-one-out method. All data were performed using R software (version 4.3.0), the R packages “TwosampleMR” (version 0.6.12) and MR-PRESSO (version 1.0).

## 3. Results

### 3.1. The results of SNPs selection and weak IVs test

In our study, we conducted MR analysis on the exposure of metformin to multiple BD studies. Tables S1–S3, Supplemental Digital Content, https://links.lww.com/MD/Q329 display detailed information on IVs of BD from multiple studies. The *F* statistics of all IVs were >10, which indicated that the results of the MR analysis were not likely to be affected by weak IVs bias.

### 3.2. The results of MR analysis

**Figure 2.**
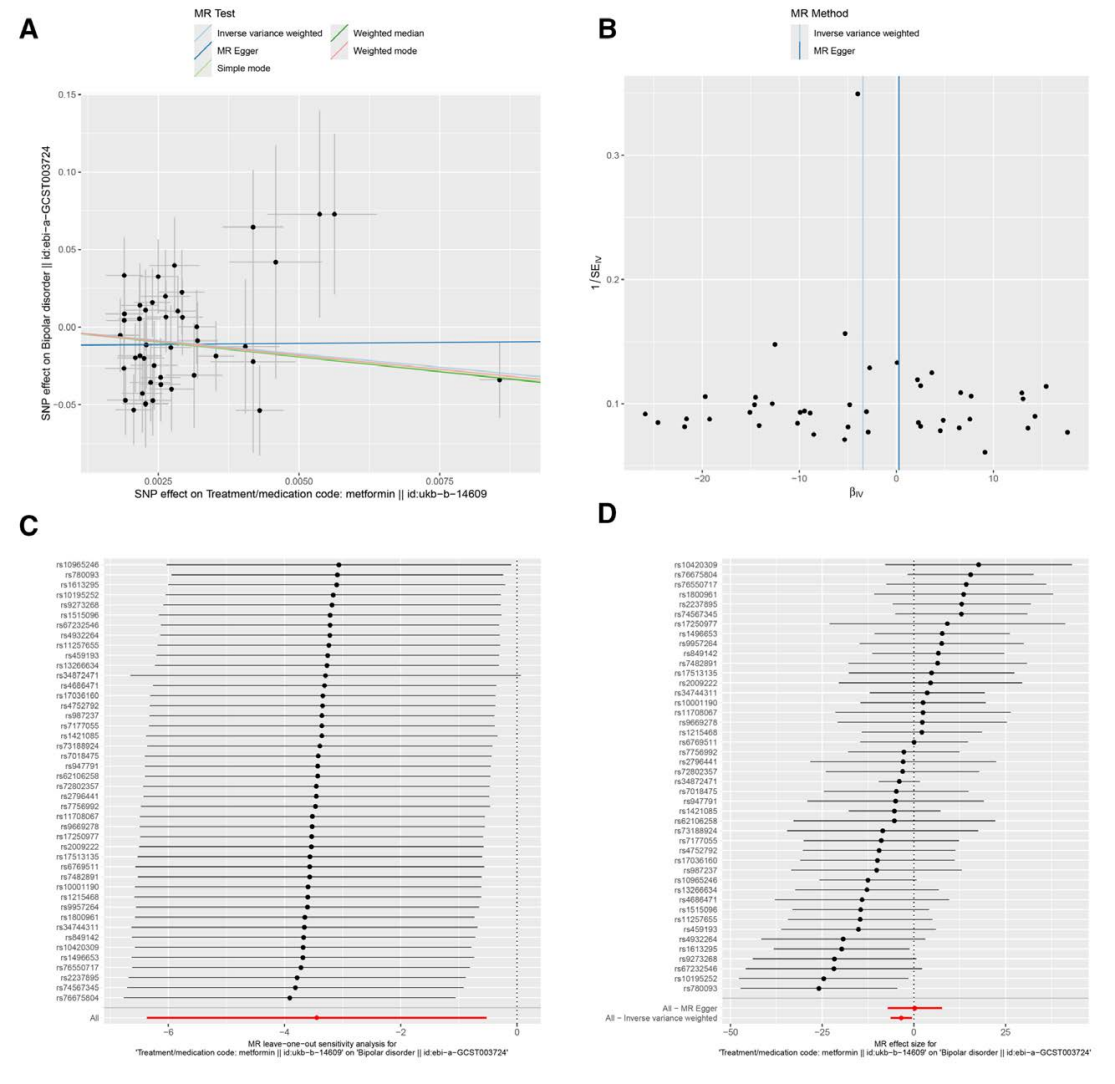
Effects of metformin on BD (ebi-a-GCST003724). (A) Scatter plot of the causal effect of metformin on BD. (B) Funnel plot of the causal effect of metformin on BD. (C) Forest plot of the leave-one-out analysis. (D) Forest plot of the causal effect of metformin on BD. BD = bipolar disorder.

**Figure 3.**
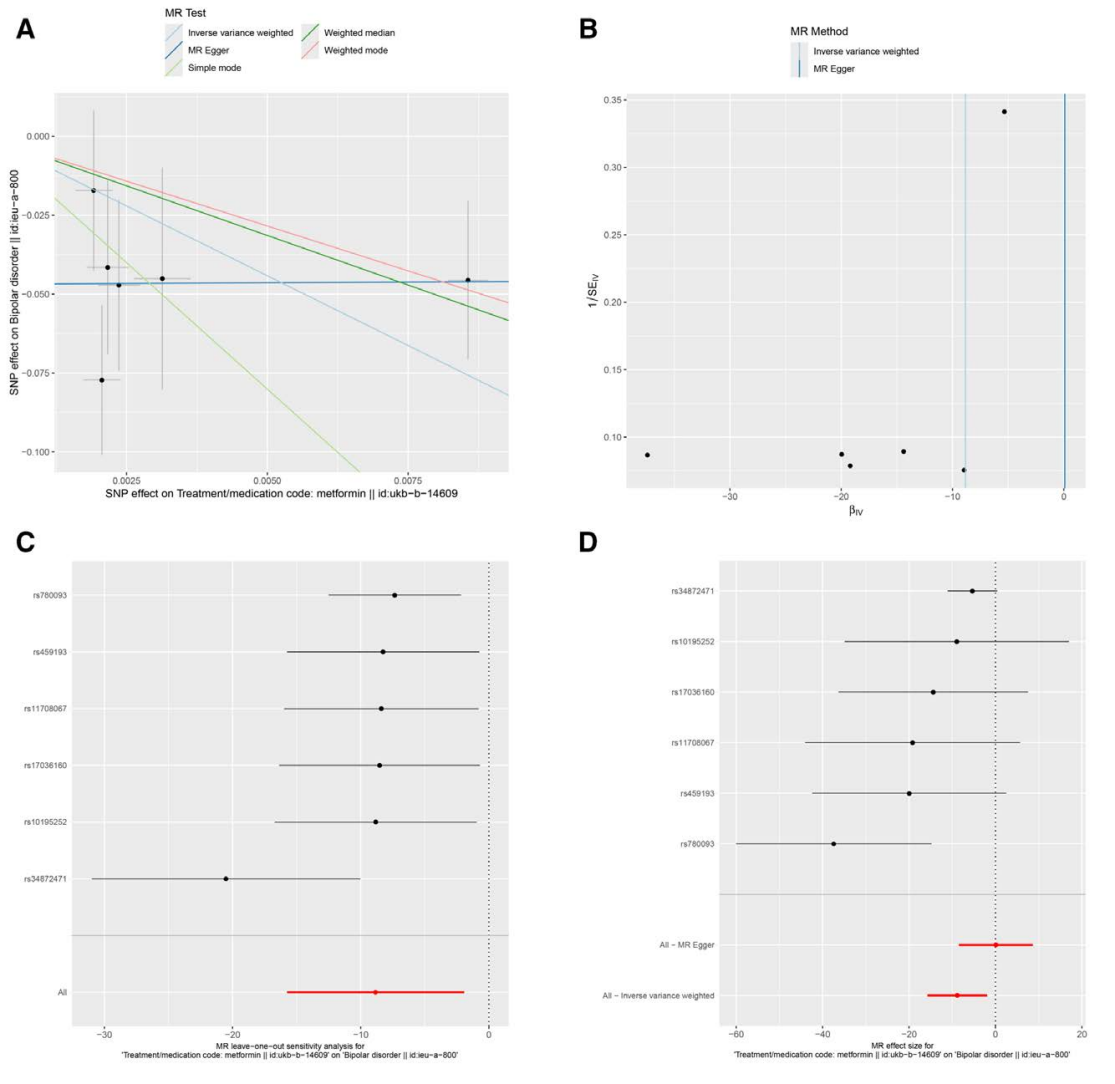
Effects of metformin on BD (ieu-a-800). (A) Scatter plot of the causal effect of metformin on BD. (B) Funnel plot of the causal effect of metformin on BD. (C) Forest plot of the leave-one-out analysis. (D) Forest plot of the causal effect of metformin on BD. BD = bipolar disorder.

**Figure 4.**
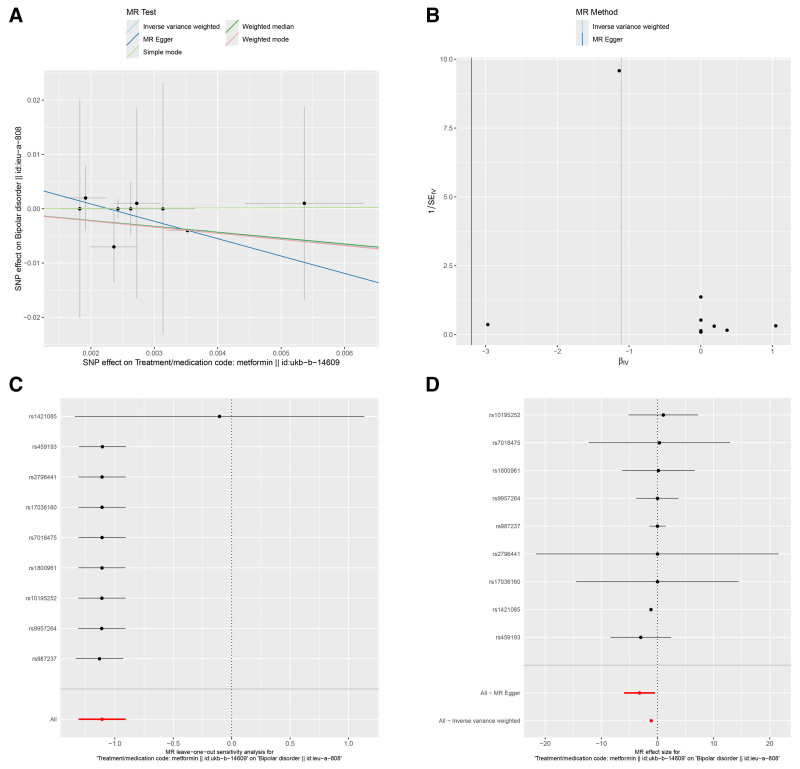
Effects of metformin on BD (ieu-a-808). (A) Scatter plot of the causal effect of metformin on BD. (B) Funnel plot of the causal effect of metformin on BD. (C) Forest plot of the leave-one-out analysis. (D) Forest plot of the causal effect of metformin on BD. BD = bipolar disorder.

In this study, we adopted MR-Egger regression, weighted median, IVW, simple mode, weighted mode from the 2-sample MR package for analysis. IVW is the most commonly used for calculating the weighted average of the impact values of all IVs, providing estimates and accuracy similar to 2-stage least squares, making the results of IVW analysis the primary focus. The IVM results indicated a causal relationship between metformin and the risk of BD. As shown in Table 2, Metformin is a protective factor for the risk of BD [odds ratio [OR] (95% confidence interval [CI]) = 0.032 (0.002–0.593), *P* = .021] (ebi-a-GCST003724, Fig. 2A), [OR (95% CI) = 1.452E‐04 (1.442E‐07 to 1.463E‐01), *P* = .012] (ieu-a-800, Fig. 3A), [OR (95%CI) = 0.33 (0.27–0.404), *P* = .000] (ieu-a-808, Fig. 4A). The IVW results showed that metformin was a protective factor in all 3 BD datasets, and the validation results were more robust for different datasets.

**Table 2:** Results of the 2-sample MR analyses.

ID exposure	ID outcome	Exposure	Outcome	Method	SNPs (n)	Beta	SE	*P*-value	OR (95% CI)
ukb-b-14609	ebi-a-GCST003724	Metformin	Bipolar disorder	MR-Egger	44	0.270	3.781	.943	1.31 (0.001–2165.123)
				Weighted median	44	‐3.834	2.347	.102	0.022 (0–2.153)
				Inverse variance weighted	44	-3.446	1.492	.021	0.032 (0.002–0.593)
				Simple mode	44	‐3.767	4.830	.440	0.023 (0–298.459)
				Weighted mode	44	‐3.624	2.704	.187	0.027 (0–5.344)
	ieu-a-800		Bipolar disorder	MR-Egger	6	0.090	4.368	.984	1.095 (0–5722.844)
				Weighted median	6	‐6.284	2.866	.028	0.002 (0–0.513)
				Inverse variance weighted	6	‐8.837	3.528	.012	0 (0–0.146)
				Simple mode	6	‐16.020	8.280	.111	0 (0–1.232)
				Weighted mode	6	‐5.684	2.866	.104	0.003 (0–0.935)
	ieu-a-808		Bipolar disorder	MR-Egger	9	-3.196	1.404	.057	0.041 (0.003–0.641)
				Weighted median	9	-1.080	0.150	.000	0.34 (0.253–0.456)
				Inverse variance weighted	9	-1.109	0.103	.000	0.33 (0.27–0.404)
				Simple mode	9	0.035	0.893	.970	1.036 (0.18–5.963)
				Weighted mode	9	-1.133	0.144	.000	0.322 (0.243–0.427)

The MR-Egger regression, weighted median, IVW, simple mode, and weighted mode were used as auxiliary analyses. The results were detailed in Table 2 and shown in Figures 2A, 3A, and 4A. Overall, these results indicated a causal relationship trend between metformin and the risk of BD, further supporting our analytical findings that metformin is a protective factor for BD.

### 3.3. The results of sensitivity analysis

The results of Cochran *Q* heterogeneity test, MR-Egger intercept test, and MR-PRESSO global test are shown in Table 3. The *P* values of Cochran *Q* were all >.05, indicating no heterogeneity. This point is further confirmed by the MR-PRESSO global test, with *P* values of .187 (ebi-a-GCST003724), 0.319 (ieu-a-800), and 0.514 (ieu-a-808), which are also insignificant. The funnel plot showed a roughly symmetrical pattern in the distribution of effect sizes, with no significant publication bias (Figs. 2B, 3B, and 4B). The leave-one-out analysis indicated that the exclusion of individual IVs had minimal impact on the results, with the overall trend remaining consistent (Figs. 2C, 3C, and 4C). Scatter plots of SNP effect sizes for metformin on BD are shown in Figures 2D, 3D, and 4D.

**Table 3:** Reliability test of MR analysis results.

ID exposure	ID outcome	Exposure	Outcome	Method	*Q*	*Q*_df	*Q*_pval	MR-Egger intercept test *P*	MR-PRESSO global test *P*
ukb-b-14609	ebi-a-GCST003724	Metformin	Bipolar disorder	MR-Egger	50.399	42	.175		
				Inverse variance weighted	51.770	43	.169	.291	.187
	ieu-a-800		Bipolar disorder	MR-Egger	3.035	4	.552		
				Inverse variance weighted	9.423	5	.093	.065	.319
	ieu-a-808		Bipolar disorder	MR-Egger	1.624	7	.978		
				Inverse variance weighted	3.846	8	.871	.180	.514

## 4. Discussion

In recent years, multiple observational studies have suggested that metformin may have a positive impact on mental health.^[24]^ In a Danish national registered study of more than 360,000 patients with type 2 diabetes, including 283,741 patients exposed to metformin, the use of metformin was associated with a significant reduction in the incidence rate of depression.^[25]^ In other studies, a case-control study of 387 women with mood disorders and 746 controls investigated the exposure to metformin and found that compared to participants who were not exposed to metformin, the use of metformin had a protective effect, reducing the likelihood of developing new mood disorders by 31%.^[26]^ However, these observational studies may be influenced by confounding factors such as lifestyle and comorbidity medication, and our MR analysis effectively reduced the interference of residual confounding and reverse causality by utilizing genetic variations as IVs, enabling us to infer potential causal effects.

This study used MR analysis to reveal for the first time a significant association between genetic prediction of metformin use and reduced risk of BD [OR (95% CI) = 0.032 (0.002–0.593), *P* = .021] (ebi-a-GCST003724, Fig. 2A), [OR (95% CI) = 0 (0–0.146), *P* = .012] (ieu-a-800, Fig. 3A), [OR (95% CI) = 0.33 (0.27–0.404), *P* = .000] (ieu-a-808, Fig. 4A). This finding suggests that metformin may serve as a potential protective factor for BD. Further supporting previous research findings that metformin may have a protective effect on mental health.^[24]^ At the same time, it also provides new theoretical basis for the application of metabolic intervention in the prevention and treatment of mental illnesses. More and more evidence suggests that patients with BD have central insulin resistance,^[27]^ and abnormalities in the insulin signaling pathway may impair neural plasticity by affecting the release of BDNF.^[28]^ Metformin, as an AMPK activator, could upregulate BDNF expression and promote synaptic remodeling.^[29]^ In addition, insulin resistance is closely related to cognitive impairment in patients with BD, and metformin may indirectly enhance cognitive function and reduce disease risk by improving glucose metabolism in the brain.^[30,31]^ Moreover, metformin enhances mitochondrial respiration through an AMPK dependent mechanism and has been shown to increase mitochondrial division in liver cells, leading to increased mitochondrial respiration and nutrient oxidation.^[32]^ Metformin can also inhibit complex I, the first enzymatic complex in the mitochondrial electron transport chain (NADH: ubiquinone oxidoreductase), thus reducing reactive oxygen species production and oxidative stress damage,^[33]^ which is highly consistent with the “mitochondrial hypothesis” of BD.^[34]^ The above research results indirectly support the potential protective effect of metformin on BD from different perspectives, further enhancing the reliability of our research results.

This study provides theoretical support for exploring the application of metformin in the prevention and adjuvant therapy of BD, especially for high-risk individuals with metabolic abnormalities such as insulin resistance and obesity, metformin may have dual benefits. The main advantage of this study lies in the use of MR method, utilizing genetic variation as an IV, effectively reducing confounding bias in traditional observational studies. In addition, we employed sensitivity analyses such as MR-Egger regression to ensure the robustness of the results. The detection results showed no heterogeneity and no significant publication bias, further supporting the effectiveness of causal inference. However, there are still several limitations to this study, the data mainly comes from populations of European descent, and the genetic structure and metabolic characteristics of BD may have racial differences, so the applicability of the conclusions in other ethnic groups needs to be carefully evaluated. MR analysis cannot provide an accurate relationship between drug dose and effect, and further verification is needed in combination with clinical randomized controlled trials in the future. Although we have employed various methods to control pleiotropy, we cannot completely rule out the possibility that certain genetic variations may affect the risk of BD through non-metformin related pathways. In addition, potential phenotype misclassification and the lack of replication in independent metformin GWAS are also limitations of our study.

## 5. Conclusion

In summary, this study provides causal evidence through MR analysis that metformin may reduce the risk of BD. This discovery opens up a new research direction for metabolic intervention strategies in psychiatric disorders and has important scientific and clinical significance, but further clinical and basic research verification is still needed.

## Author contributions

**Conceptualization:** Yichuan Lin.

**Data curation:** Zhitao Li.

**Formal analysis:** Zhitao Li, Lili Wang, Xinyu Sun.

**Funding acquisition:** Lili Wang.

**Investigation:** Zhitao Li.

**Methodology:** Zhitao Li, Lili Wang, Xinyu Sun.

**Project administration:** Zhitao Li.

**Resources:** Zhitao Li, Yichuan Lin.

**Software:** Zhitao Li.

**Supervision:** Yichuan Lin.

**Writing – original draft:** Zhitao Li.

**Writing – review & editing:** Yichuan Lin.

## Supplementary Material


